# Dielectric Metasurface for Generating Longitudinally Separated Dual-Channel Focused Vectorial Structured Light

**DOI:** 10.3390/nano16070389

**Published:** 2026-03-24

**Authors:** Haoyan Zhou, Xinyi Jiang, Wenxin Wang, Yuantao Wang, Yuchen Xu, Kaixin Zhao, Chuanfu Cheng, Chunxiang Liu

**Affiliations:** Shandong Provincial Key Laboratory of Light Field Manipulation Physics and Applications & School of Physics and Optoelectronics, Shandong Normal University, Jinan 250014, China; zhouhaoyanrock@163.com (H.Z.); 19735201452@163.com (X.J.); 19854190974@163.com (W.W.); wangyuantao0210@163.com (Y.W.); 15628848390@163.com (Y.X.); chengchuanfu@sdnu.edu.cn (C.C.)

**Keywords:** metasurface, vector beams, longitudinal manipulation, multi-channel

## Abstract

The manipulation of vector beams (VBs) with longitudinally variant polarization states is an important research topic and has potential applications in classical and quantum fields. In this study, we propose a half-wave plate dielectric metasurface composed of two interleaved sub-metasurfaces to generate longitudinally separated dual-channel vectorial structured light fields. The propagation and Pancharatnam–Berry phases are employed to construct hyperbolic, helical, and opposite gradient phases for focusing wavefronts, generating circularly polarized (CP) vortices, and deflecting CP vortices with the same chirality in opposite directions. Consequently, dual-channel higher-order or hybrid-order Poincaré (HOP or HyOP) beams are generated along the optical axis under elliptically polarized illumination, and their polarization states evolve along an arbitrary pair of antipodal meridians on the HOP or HyOP sphere by varying the ellipticity of the incident light, the propagation-phase topological charge, and the rotation order of the meta-atom. The consistency between the theoretical and simulated results demonstrates the feasibility and practicability of the proposed method. This study is significant for compact, integrated, and multifunctional optical devices, and provides an innovative strategy to extend optical field manipulation from two-dimensional to three-dimensional space.

## 1. Introduction

Polarization and phase are two crucial properties of light. Each photon in light can possess a spin angular momentum (SAM) of *σ*ℏ and an orbital angular momentum (OAM) of *l*ℏ based on the chirality of circular polarization and the helical phase structure, respectively [1,2,3]. Vector beams (VBs), as typical vectorial structured light, have attracted extensive attention and found applications in various fields from classical physics to quantum science owing to the distinctive characteristics of inhomogeneous polarization distribution. The important classical applications include optical trapping [4], high-resolution microscopy [5], and optical encryption and communication [6,7]. In quantum science, VBs have been used for quantum communication [8], quantum walks [9], and quantum key distribution [10]. Explorations of VBs have facilitated the discoveries of various interesting phenomena in light fields, including Möbius strips [11], the sub-diffraction focusing spot, and knotted structures in vector and polarization fields [12,13]. Milione et al. introduced the concept of a high-order Poincaré sphere (HOP) to describe the OAM, state of polarization, and phase profile of VBs [2]. The two poles of the HOP sphere represent left- and right-handed circularly polarized (LCP and RCP) vortices with topological charges of the same absolute value (|*l*_LCP_| = |*l*_RCP_|). Each point on the HOP sphere corresponds to a spatially variant polarized light field generated by the weighted superposition of the two orthogonal vortices [14]. To characterize the evolution of polarization states and phases in inhomogeneous anisotropic media and extend the applicability of the HOP sphere, a hybrid-order Poincaré sphere (HyOP) has been proposed, wherein the north and south poles correspond to LCP and RCP vortices with distinct topological charges (|*l*_LCP_| ≠ |*l*_RCP_|) [15]. However, conventional macroscopic HOP and HyOP beams are typically generated by bulky optical devices, such as spatial light modulators [16], spiral phase plates [17,18], q-plates [19], and Dammann gratings [20], which renders their compliance with the requirements of miniaturization, integration, and multifunctionality impractical [21].

Based on the generalized Fresnel theorem and the abrupt phase shifts induced by meta-atoms [22], metasurfaces enable local manipulation of the amplitude, phase and polarization of light at the subwavelength scale, and have been successfully developed for diverse applications, such as metalenses [23], optical encryption and anti-counterfeiting [24], on-chip integration [25], and multichannel holographic imaging [26]. Specifically, among all the fascinating applications, metasurfaces have become an effective tool for generating multiplexed focused vortex and vector beams. Liu et al. designed a spin-multiplexed metasurface that enables the generation of broadband perfect Poincaré beams in the visible light band [27]. Jin et al. proposed a geometric phase-based metasurface to realize the generation of an angle multiplexed multichannel optical vortex array [21]. Ahmed et al. designed a double-layer geometric metasurface to achieve the superposition of grafted perfect vortex beams in multiple channels [28]. However, previous studies have primarily focused on the generation of vectorial structured light within a single transverse plane perpendicular to the direction of light propagation.

The longitudinal polarization variation adds a new dimension to field applications, and using propagation distance as an extra degree of freedom extends light-field manipulation from 2D to 3D, thus expanding the application potential of metasurfaces in optical communication, light-matter interaction, and laser processing. Luo et al. designed a circularly polarized multiplexed dielectric metasurface with spin-decoupled isotropic meta-atoms for independent phase channel modulation, enabling two-channel longitudinal scalar-vector beam conversion [29]. Yang et al. proposed an all-dielectric metasurface based on axicon phase and spiral phases, and realized the generation of arbitrary vector Bessel beams with high OAM mode purity on the HOP sphere [30]. Luo et al. demonstrated the generation of structured terahertz beams with longitudinally varied polarization from linearly polarized (LP) incidence by designing an anisotropic metasurface combining dynamic and geometric phases [31]. Yang et al. realized the generation of self-evolving vector Bessel beams via proposed metasurfaces by modulating the polarization state of incident light [32]. Previous metasurface studies have primarily focused on generating beams with identical topological charges for LCP and RCP components. Moreover, although propagation distance tuning has been incorporated into metasurface design, its application has been limited to the generation of conventional HOP beams. Therefore, it could be a challenging research subject to design a metasurface for generating longitudinally separated multi-channel vectorial structured light.

In this study, we propose a half-wave plate (HWP) dielectric metasurface for generating longitudinally separated dual-channel vectorial structured light fields, which is composed of two sub-metasurfaces, M_A_ and M_B_. The propagation phase is employed to construct the hyperbolic phase *φ_lens_* and helical phase $\varphi_{he,\;p}^{A/B}$, while the Pancharatnam–Berry (P-B) phase is used to construct the helical phases $\varphi_{he,\;g}^{A/B}$ and the opposite radial gradient phase *φ_ra_* for the two sub-metasurfaces. Under illumination with elliptically polarized light, the cross-polarization components with the same chirality from the two sub-metasurfaces are deflected in opposite directions, and two pairs of vortices with opposite topological charges are subsequently focused on two channels, *z*_1_ and *z*_2_, along the optical axis, respectively. Therefore, longitudinally separated dual-channel vectorial structured lights are generated by the superposition of two orthogonal circularly polarized vortices. We performed theoretical analysis and simulated the output light field using the finite-difference time-domain (FDTD) method. This study advances the development of multifunctional integrated optical devices and holds significant implications for optical micromanipulation, light-matter interaction, as well as classical and quantum communications.

## 2. Principle of Metasurface Design

### 2.1. Overview of Principle

**Figure 1:**
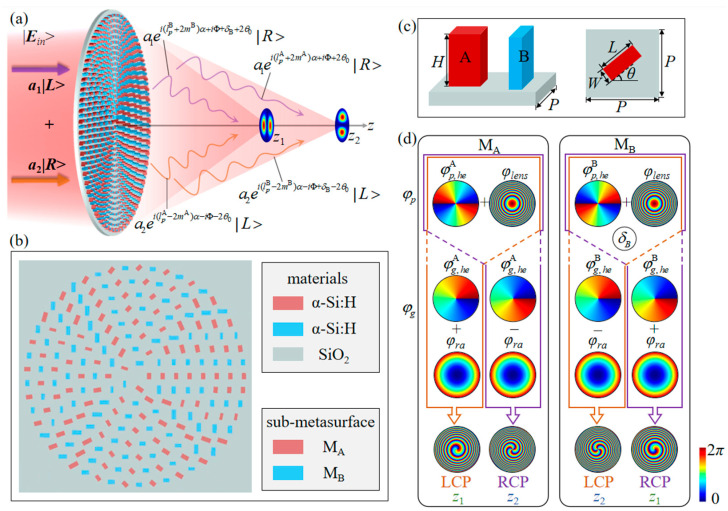
Schematic of the design principles for the metasurface. (**a**) Schematic illustration of generating dual-channel vectorial structured light fields along the optical axis under elliptically polarized illumination. (**b**) The top view of the local region at the center of the metasurface. (**c**) The side and top views of the meta-atoms. (**d**) The phase profiles of the sub-metasurfaces M_A_ and M_B_, respectively.

Figure 1a schematically illustrates the generation of longitudinally separated dual-channel vectorial structured light fields at two focal planes, *z*_1_ and *z*_2,_ using an HWP dielectric metasurface under elliptically polarized illumination. Figure 1b shows a top-view schematic of the local region at the center of the metasurface. The proposed metasurface is configured with two interleaved sub-metasurfaces, M_A_ and M_B_. Each sub-metasurface is composed of meta-atoms arranged on concentric circular rings, with both the azimuthal and radial spacing equal to the lattice constant *P* = 380 nm. For the arrangement, the azimuthal position α = 0° of each concentric ring is taken as the starting point. Meta-atoms located at arc lengths of odd multiples of *P* on each ring are assigned to sub-metasurface M_A_, and those at even multiples of *P* belong to sub-metasurface M_B_. Meta-atoms A and B are rectangular nanopillars of α-Si:H on a SiO_2_ substrate, and their side and top views are shown in Figure 1c, where *H*, *L*, *W*, *θ*, and *P* represent the height, length, width, orientation angle, and lattice constant of the meta-atoms, respectively. The height *H* of the nanopillars remains constant. To generate the longitudinally separated dual-channel vectorial structured light field, the propagation and P-B phase are utilized to construct hyperbolic, helical, and gradient phases. Figure 1d presents the phase profiles imparted to the transmitted light field of the two sub-metasurfaces. The propagation phase is employed to construct a hyperbolic phase *φ_lens_* for beam focusing and a helical phase $\varphi_{p,\;he}^{A/B}$ for vortex generation, respectively. Moreover, an additional phase factor *δ*_B_ is introduced for the sub-metasurface M_B_ to modulate the phase difference between the two orthogonal circular polarization (CP) vortices in each channel.

(1) $$\varphi_{p}^{A}=\varphi_{lens}+\varphi_{p,\;he}^{A}=-k(\sqrt{r^{2}+f^{2}}-f)+l_{p}^{A}\alpha$$

(2) $$\varphi_{p}^{B}=\varphi_{lens}+\varphi_{p,\;he}^{B}=-k(\sqrt{r^{2}+f^{2}}-f)+\delta_{B}+l_{p}^{B}\alpha$$

where *k* = 2*π*/*λ* denotes the wave number, *f* is the focal length of the metasurface, and *α* is the azimuthal angle of the meta-atom position. The P-B phase is used to construct opposite radial gradient phases $\varphi_{ra}^{A/B}$ for M_A_ and M_B_, enabling two orthogonal CP components from each sub-metasurface to split in the opposite direction along the optical axis. Meanwhile, the P-B phase is also utilized to construct a helical phase $\varphi_{g,\;he}^{A/B}$.

(3) $$\varphi_{g}^{A/B}=\varphi_{ra}^{A/B}+\varphi_{g,\;he}^{A/B}=\sigma \tau^{A/B}k(\sqrt{r^{2}+f^{2}}-\sqrt{r^{2}+{\left(f+\Delta \right)}^{2}}+\Delta )+2\sigma m^{A/B}\alpha$$

where *σ* = 1 and *σ* = −1 represent the RCP and LCP components of the transmitted light field, respectively. The coefficients *τ*^A^ = 1 and *τ*^B^ = −1 correspond to the radial gradient phases of M_A_ and M_B_, respectively, ∆ is the spin separation distance, and *m* is the rotation order of the meta-atom, and the vortex topological charges of the two sub-metasurfaces satisfy $l_{g}^{A/B}=2\sigma m^{A/B}$.

Under LCP illumination, the opposite radial gradient phases imparted to the sub-metasurfaces M_A_ and M_B_ cause their cross-polarized components to focus on the focal plane *z*_2_ and *z*_1_, respectively, as shown in the purple box in Figure 1d. When RCP light is incident, the cross-polarized components of M_A_ and M_B_ depicted by the orange box are focused at *z*_1_ and *z*_2_, respectively. Therefore, under elliptically polarized illumination, the superposition of the cross-components of LCP and RCP vortices from sub-metasurface M_A_ and M_B_ forms a VB at the focal plane *z*_1_. In contrast, at the focal plane *z*_2_, another VB is generated by the superposition of the cross-components of RCP and LCP vortices from M_A_ and M_B_. By adjusting the ellipticity of the incident elliptically polarized light, the amplitude ratio of LCP to RCP vortices in each channel is varied, such that the polarization states of the generated dual-channel vectorial structured light field correspond to the points on a meridian of HOP or HyOP sphere. When $l_{p}^{A/B}=0$, *m*^A^ = *m*^B^, and the LCP and RCP vortices generated by the two sub-metasurfaces carry the topological charges of −2*m*^A^ and 2*m*^A^, respectively. Thus, two orthogonal vortices with equal topological charge but opposite signs are superposed to form HOP beams in each channel. Moreover, if the additional phase factor *δ*_B_ is set to *π*/2 and the initial orientation angle *θ*_0_ of the meta-atom is adjusted to a suitable value, the polarization states of the generated dual-channel VBs evolve along the prime meridian and the anti-meridian of the HOP sphere, respectively. In contrast, when $l_{p}^{A/B}\neq 0$, HOP or HyOP beams of different orders can be generated in the two channels. Overall, the dual-channel vectorial structured light fields with polarization states evolving along any two arbitrary meridians on the HOP or HyOP spheres can be generated by modulating the propagation and P-B phases, as well as the ellipticity and polarization angle of the incident light.

### 2.2. Theoretical Analysis

Each meta-atom can be regarded as an anisotropic polarization element that functions as an arbitrary wave plate. The fast and slow axes are parallel and perpendicular to the long side direction of the meta-atom, respectively. When the orientation angle of the meta-atom is *θ*, the Jones matrix can be expressed as

(4) $$J(x,\;y)=R(-\theta )\left[\begin{array}{cc} t_{xx}e^{i\varphi_{xx}(x,\;y)} & 0\\ 0 & t_{yy}e^{i\varphi_{yy}(x,\;y)} \end{array}\right]R(\theta )$$

where R(*θ*) is a 2 × 2 rotation matrix. *t_xx_* and *t_yy_* represent the amplitudes of the transmitted light field along the fast and slow axes, respectively, which are approximately equal. *φ_xx_*(*x*, *y*) and *φ_yy_*(*x*, *y*) are the phases of the transmitted light field, Δ*φ* = |*φ_yy_*(*x*, *y*) − *φ_xx_*(*x*, *y*)| is the phase retardation, and the propagation phase of the meta-atom is *φ_p_*(*x*, *y*) = *φ_xx_*(*x*, *y*). When elliptically polarized light is incident on a half-wave plate, we obtain the following:

(5) $$J(x,\;y)|L\rangle =e^{i\varphi_{1}(x,\;y)}|R\rangle$$

(6) $$J(x,\;y)|R\rangle =e^{i\varphi_{2}(x,\;y)}|L\rangle$$

The right-hand sides of the equations represent the cross-polarized components with phases *φ*_1_(*x*, *y*) and *φ*_2_(*x*, *y*), which can be jointly modulated by the propagation phase *φ_p_*(*x*, *y*) and the P-B phase *φ_g_*(*x*, *y*) = 2*σθ*(*x*, *y*). Thus, the Jones matrix of an HWP meta-atom can be written as follows:

(7) $$J(x,y)=\left[\begin{array}{cc} e^{i\varphi_{1}(x,y)} & e^{i\varphi_{2}(x,y)}\\ -ie^{i\varphi_{1}(x,y)} & ie^{i\varphi_{2}\left(x,y\right)} \end{array}\right]{\left[\begin{array}{cc} 1 & 1\\ i & -i \end{array}\right]}^{-1}$$

Based on the eigenvalues and eigenvectors of this matrix, we can derive the propagation phases *φ_xx_*(*x*, *y*) and *φ_yy_*(*x*, *y*), as well as the orientation angle *θ*(*x*, *y*) of the meta-atom, as follows:

(8) $$\varphi_{xx}(x,y)=[\varphi_{1}(x,y)+\varphi_{2}(x,y)]/2$$

(9) $$\varphi_{yy}(x,y)=[\varphi_{1}(x,y)+\varphi_{2}(x,y)]/2+\pi$$

(10) $$\theta (x,y)=\;[\varphi_{1}(x,y)-\varphi_{2}(x,y)]/4$$

The above equations provide a quantitative relationship for determining the propagation phases and orientation angle of the meta-atom based on the target phases *φ*_1_(*x*, *y*) and *φ*_2_(*x*, *y*). The condition |*φ_xx_*(*x*, *y*) − *φ_yy_*(*x*, *y*)| = *π* indicates that the meta-atom exhibits the characteristics of an HWP.

As is well known, elliptically polarized light can be represented by a polarization state corresponding to a point (2Θ’, 2Φ’) on the conventional Poincaré sphere (PS), expressed as a superposition of LCP and RCP components, i.e., ***E***_in_ = *a*_1_*e*^-^*^i^*^Φ’^[1 *i*]^T^ + *a*_2_*e^i^*^Φ’^[1 − *i*]^T^. Here, the coefficients *a*_1_ = sinΘ’ and *a*_2_ = cosΘ’ are the normalized amplitudes of the LCP and RCP components, respectively. Substituting Equations (1)–(3) into Equations (5) and (6), we can obtain that the dual-channel vectorial structured light fields generated at the two focal planes *z*_1_ and *z*_2_ are expressed as follows:

(11) $$E_{1}=C_{1}(a_{2}e^{i(l_{p}^{A}-2m^{A})\alpha -i\Phi -2\theta_{0}}\left[\begin{array}{c} 1\\ i \end{array}\right]+a_{1}e^{i(l_{p}^{B}+2m^{B})\alpha +i\Phi +\delta_{B}+2\theta_{0}}\left[\begin{array}{c} 1\\ -i \end{array}\right])$$

(12) $$E_{2}=C_{2}(a_{2}e^{i(l_{p}^{B}-2m^{B})\alpha -i\Phi +\delta_{B}-2\theta_{0}}\left[\begin{array}{c} 1\\ i \end{array}\right]+a_{1}e^{i(l_{p}^{A}+2m^{A})\alpha +i\Phi +2\theta_{0}}\left[\begin{array}{c} 1\\ -i \end{array}\right])$$

where $C_{1}=e^{ik[-\sqrt{r^{2}+{\left(f+\Delta \right)}^{2}}+f+\Delta ]}$ and $C_{2}=e^{-ik(2\sqrt{r^{2}+f^{2}}-\sqrt{r^{2}+{\left(f+\Delta \right)}^{2}}+f+\Delta )}$. From Equations (11) and (12), we can see that an LCP vortex with topological charge $l_{p}^{A}-2m^{A}$ and an RCP vortex with topological charge $l_{p}^{B}+2m^{B}$ are generated on the focal plane *z*_1_, while an LCP vortex with topological charge $l_{p}^{B}-2m^{B}$ and an RCP vortex with topological charge $l_{p}^{A}+2m^{A}$ are formed on the focal plane *z*_2_. By controlling the topological charge $l_{p}^{A/B}$ and the rotation order *m*^A/B^, we can simultaneously obtain HOP beams of the same order, HOP beams of different orders, and HyOP beams of different orders on the two focal planes. Meanwhile, the weights of the LCP and RCP vortices on the two focal planes can be adjusted by tuning the ellipticity of the incident light. The phase difference between the two orthogonal CP vortices can be modulated by the angle Φ’ of the incident light, the initial orientation angle *θ*_0_, and the additional phase factor *δ*_B_. Specifically, when *δ*_B_ = 0 and *δ*_B_ = *π*, the phase differences between the two orthogonal vortices on two focal planes, *z*_1_ and *z*_2_, are equal. When *δ*_B_ = *π*/2, these phase differences differ by *π*. In this case, varying the ellipticity of the incident light causes the polarization states of the dual-channel vectorial structured light fields to evolve along two meridians separated by 180° in longitude on the HOP sphere. Therefore, the proposed metasurface in this work enables the arbitrary manipulation of the polarization states of HOP or HyOP beams.

## 3. Simulation Results

**Figure 2:**
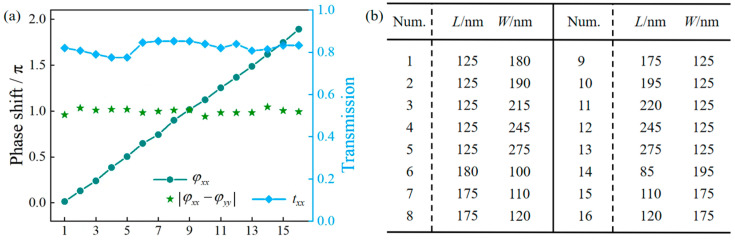
(**a**) The propagation phase *φ_xx_*(*x*, *y*), the phase retardation |*φ_xx_*(*x*, *y*) − *φ_yy_*(*x*, *y*)|, and the transmission amplitude *t_xx_* in the *x*-direction versus the serial number *N* of the HWP meta-atoms. (**b**) Dimensions of the sixteen selected meta-atoms.

To verify the feasibility of the metasurface designed in this paper, we first conduct theoretical simulations using MATLAB R2014b, and then perform simulation calculations on the transmitted light field using the FDTD software (Lumerical 2020 R2). The meta-atom is a rectangular α-Si:H nanopillar with a height of 480 nm, an orientation angle *θ*, and a lattice constant *P* of 380 nm, fabricated on a SiO_2_ substrate. At an incident wavelength of *λ* = 800 nm, the refractive index *n* of the meta-atom is 3.744, and the extinction coefficient *κ* is 0.000. We perform parameter sweeps on a single meta-atom under linearly polarized illumination along the *x*- and *y*-directions. The side length of the nanopillar is scanned from 80 nm to 330 nm with a step size of 1 nm. The boundary condition along the direction parallel to the polarization of the incident light is set as “Anti-symmetric”, while that perpendicular to the polarization direction within the *xOy* plane is set as “Symmetric”, and the *z*-direction boundary is set as “Perfectly Matched Layer (PML)”. Thus, we can obtain the phases *φ_xx_*(*x*, *y*) and *φ_yy_*(*x*, *y*), as well as transmission amplitudes *t_xx_* and *t_yy_* of meta-atoms with different sizes under linearly polarized incidence along the *x*- and *y*-directions. Firstly, we selected meta-atoms with the phase retardation |*φ_xx_*(*x*, *y*) − *φ_yy_*(*x*, *y*)| in the range of 0.9*π* to 1.1*π* to ensure they exhibit ideal HWP performance. Secondly, we further selected a group of meta-atoms whose propagation phase *φ_xx_*(*x*, *y*) increases linearly and uniformly from 0 to 2*π*, i.e., the propagation phase of the N-th meta-atom must satisfy *φ_xx,_*_N_ = (N − 1)*π*/8. Finally, the transmission amplitudes *t_xx_* and *t_yy_* of the meta-atom should be approximately equal and greater than 0.8 to generate high-quality vortex beams with uniform intensity distribution. Through this rigorous selection process, sixteen meta-atoms fully satisfying the design requirements were chosen from the parameter sweep results. Figure 2a presents the propagation phase *φ_xx_*(*x*, *y*), phase retardation |*φ_xx_*(*x*, *y*) − *φ_yy_*(*x*, *y*)|, and transmission amplitude *t_xx_* of these sixteen meta-atoms. The specific dimensions of these meta-atoms are shown in Figure 2b. Notably, in the parameter sweeps conducted via the FDTD method, the transmission phases *φ_xx_* and *φ_yy_* are obtained, respectively, under the incidence of *x*- and *y*-polarized light. From the symmetry, the transmission phases *φ_xx_* and *φ_yy_* would be identical under the exchange of the *x* and *y* axes [33]. This indicates that the propagation phase difference between two such meta-atoms with swapped length and width is approximately *π*.

We designed a metasurface sample consisting of sixteen selected meta-atoms periodically arranged on concentric rings. The starting position for each concentric ring was defined at azimuthal angle *α* = 0, and the meta-atoms were alternately assigned to the two sub-metasurfaces. Specifically, meta-atoms at odd- and even-numbered positions formed sub-metasurfaces M_A_ and M_B_, respectively, as illustrated in Figure 1a. By substituting the coordinates of each meta-atom into Equations (1)–(3), we obtained the required size and orientation angle for the corresponding position and implemented the spatial arrangement. The spacing between adjacent meta-atoms on the same ring and the spacing between adjacent rings were both set to a lattice constant *P* = 380 nm. In the simulations, the interface between the SiO_2_ substrate and the meta-atoms was defined as *z* = 0 μm. The incident light illuminated the metasurface from a plane one wavelength (*λ* = 800 nm) below the upper surface of the SiO_2_ substrate (*z* = −0.8 μm). A monitor plane was placed at a distance of one wavelength from the bottom surface of the meta-atom (*z* = 0.8 μm) to record the near-field light field distribution. Then, by projecting the near-field data to the desired far-field region, the light field distributions on the focal planes *z*_1_ and *z*_2_ were obtained. All parameters were set according to our previous experimental work [34,35,36,37]. In this paper, three metasurfaces were designed. For the first metasurface M_1_, the propagation-phase topological charge was set to $l_{p}^{A/B}=0$, the meta-atom rotation order *m*^A/B^ = 0.5, the focal length *f* = 150 μm, the spin separation distance Δ = 20 μm, the additional phase factor *δ*_B_ = *π*/2, the initial orientation angle *θ*_0_ = −*π*/8, and the diameter of the sample was 76 μm. When LP light was incident, the LCP and RCP components generated by the two sub-metasurfaces carried vortices of orders −1 and 1, respectively. Consequently, the first-order VBs were generated on the two focal planes, and the phase differences between the two orthogonal CP vortices were 0 and *π*, respectively, corresponding to radially and azimuthally polarized VBs (RPVBs and APVBs). Meanwhile, by illuminating the metasurface with elliptically polarized light of different ellipticities, the weights of the LCP and RCP vortices can be modulated. Therefore, the polarization states of the dual-channel HOP beams exhibit evolution trajectories along the prime meridian (2Φ = 0°) and the antimeridian (2Φ = 180°) of the HOP sphere. Based on the above design, we simulated the transmitted light field of the metasurface using FDTD software. During the simulation, the boundary conditions in the *x*, *y*, and *z* directions were all set as PML. The upper-surface position of the metasurface substrate, the incident light position, and the monitoring plane were set to *z* = 0, *z* = −0.8 µm, and *z* = 0.8 µm, respectively. The incident wavelength was set to *λ* = 800 nm. The FDTD simulation region spanned 77 µm × 77 µm in the *x-* and *y*-directions, and extended from −0.9 µm to 1.1 µm along the propagation direction, encompassing the metasurface sample, the incident light, and the monitoring plane. The mesh type was set to auto non-uniform, and the mesh accuracy was set to 1. During the simulation, we first simulated the near-field light distribution of the metasurface sample under incident light with different ellipticities, and then projected it to the far-field plane to obtain the generated vectorial structured light fields on the two focal planes.

**Figure 3:**
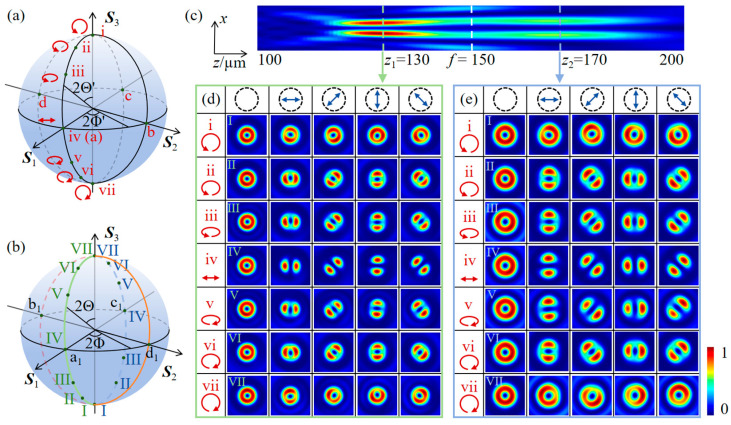
(**a**) Schematic of elliptically polarized incidence mapped onto the conventional PS. (**b**) Polarization states of the corresponding VBs generated in the two channels corresponding to points on the HOP sphere. (**c**) The intensity images of the longitudinally separated dual-channel HOP beams generated on the *xOz* plane under the illumination of *x*-LP light. The total intensity and *x*-, 45°, *y*-, and 135° component intensity patterns formed on (**d**) the *z*_1_ focal plane and (**e**) the *z*_2_ focal plane under different elliptical polarization illuminations.

Figure 3a schematically displays the polarization states of the elliptically polarized light. The labeled points i to vii correspond to polarization states at latitudes of 2Θ’ = 0°, 30°, 45°, 90°, 135°, 150°, and 180°, respectively, on the prime meridian of the conventional PS. Figure 3b shows the polarization states of the dual-channel HOP sphere generated on the focal planes *z*_1_ and *z*_2_ under illumination with different ellipticities, corresponding to the polarization states labeled as points I to VII on the prime meridian (green line) and the antimeridian (blue line). Figure 3c shows the intensity patterns of LCP and RCP vortices on the *xOz* plane, demonstrating that the dual-channel VBs are generated by the sample M_1_ under horizontally LP illumination. The *z*-axis spans from 100 µm to 200 µm. The two focal planes at *z*_1_ = 130 µm and *z*_2_ = 170 µm are marked by green and blue dashed lines, respectively. The white dashed line denotes the focal length *f* = 150 µm of the metasurface. Figure 3d and Figure 3e show the simulated intensity distributions of the total and component fields on the focal planes *z*_1_ and *z*_2_ generated by M_1_ under different elliptical polarization illuminations, respectively. The top row labels from left to right denote the total intensity, *x*-, 45°, *y*-, and 135° polarized components. The red arrows in the leftmost column indicate the elliptical polarization of the incident light, corresponding to the points on the conventional PS shown in Figure 3a. Under horizontally LP illumination, each component intensity exhibits a highly symmetric petal-like pattern completely separated by dark lines, and the number of petals is twice the absolute value of the vortex topological charge. Due to the nonseparability of polarization and spatial mode of VBs, the orientation of the dark lines rotates with the transmission axis of the polarizer. The dark lines in Figure 3d,e are respectively perpendicular and parallel to the transmission axis, indicating that the RPVBs and APVBs are generated on focal planes *z*_1_ and *z*_2_, respectively, under horizontally LP illumination. With increasing ellipticity of the incident light, the orientation of the dark line in each component pattern remains unchanged, whereas the dark line gradually becomes blurred as the ellipticity decreases and disappears completely under CP incidence, with the intensity profile exhibiting a doughnut-shaped pattern. The polarization states of the VBs generated on the focal planes *z*_1_ and *z*_2_ evolve along the prime meridian (green line) and the antimeridian (blue line) of the HOP sphere, respectively, with variation in the incident ellipticity, as illustrated in Figure 3b.

**Figure 4:**
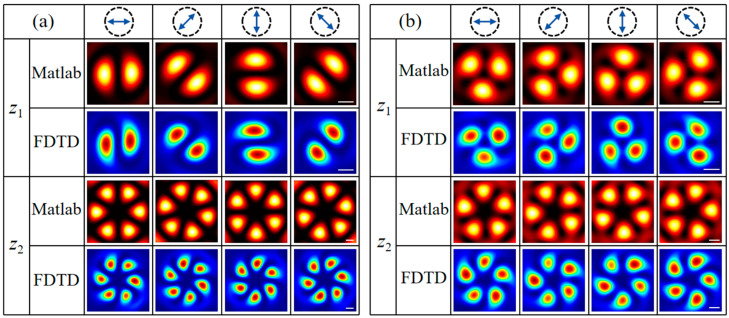
Intensity patterns of the dual-channel (**a**) HOP beams and (**b**) HyOP beams under horizontally LP illumination. Each column from left to right corresponds to the *x*-, 45°, *y*-, and 135° components of the transmitted field on the focal plane *z*_1_ and *z*_2_, respectively.

Furthermore, the designed metasurface can also generate more complex vectorial structured light fields. Figure 4 presents the simulated intensity images of dual-channel HOP and HyOP beams of different orders on the two focal planes, *z*_1_ and *z*_2_, under horizontally LP illumination, obtained using the MATLAB software and FDTD method. The solid white line in the figure indicates the scale bar, corresponding to a length of 1 µm. Metasurface sample M_2_ is designed to generate dual-channel HOP beams of different orders. Specifically, the propagation-phase topological charges are $l_{p}^{A}=2$ and $l_{p}^{B}=-2$, the meta-atom rotation orders are *m*^A/B^ = 0.5, the focal length is *f* = 150 μm, the spin separation distance is Δ = 20 µm, the additional phase factor is *δ*_B_ = 0, the initial orientation angle is *θ*_0_ = 0, and the sample diameter is 114 µm. Thus, an LCP vortex with topological charge *l*_LCP_ = 1 from sub-metasurface M_A_ and an RCP vortex with *l*_RCP_ = −1 from M_B_ are generated on the focal plane *z*_1_; while on the focal plane *z*_2_, an RCP vortex with *l*_RCP_ = 3 from M_A_ and an LCP vortex with *l*_LCP_ = −3 from M_B_ are formed. Consequently, the superposition of two pairs of orthogonal CP vortices in each channel leads to the generation of first-order *π*-RPVB and third-order RPVB on the focal planes *z*_1_ and *z*_2_ under horizontally LP illumination, as shown in Figure 4a. Metasurface sample M_3_ is designed to generate dual-channel HyOP beams. In particular, the topological charges and rotation orders of the two sub-metasurfaces are set to $l_{p}^{A}=2$, $l_{p}^{B}=-2$, *m*^A^ = 0.5, and *m*^B^ = 0. The other parameters remain identical to those of metasurface M_2_. In this case, M_A_ generates an LCP vortex with *l*_LCP_ = 1 and an RCP vortex with *l*_RCP_ = 3 on the focal planes *z*_1_ and *z*_2_, respectively; while M_B_ generates an RCP vortex with *l*_RCP_ = −2 and an LCP vortex with *l*_LCP_ = −2 on the focal planes *z*_1_ and *z*_2_, respectively. The superposition of |1>+|−2> on *z*_1_ and |−2>+|3> on *z*_2_ causes the generation of dual-channel HyOP beams under elliptically polarized illumination. Figure 4b shows the corresponding simulated results under horizontally LP illumination.

**Figure 5:**
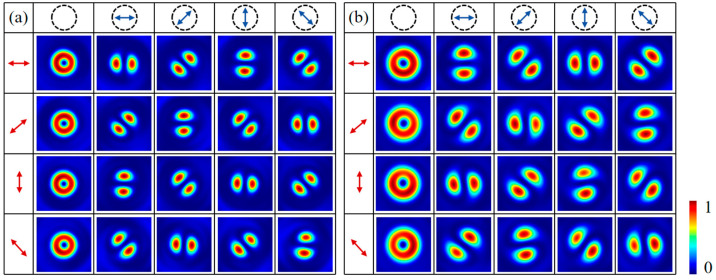
Intensity patterns of dual-channel HOP beams generated by metasurface M_1_ on the two focal planes (**a**) *z*_1_ and (**b**) *z*_2_ under LP illumination with different polarization angles.

Furthermore, the phase difference between the two orthogonal CP vortices in each channel can be modulated by changing the polarization angle Φ’ of the incident light, enabling the generation of VBs on different meridians of the HOP sphere. Figure 5 shows the intensity patterns of the total field, *x*-, 45°, *y*-, and 135° components generated by metasurface M_1_ under the LP illumination with the polarization angles of 0°, 45°, 90°, and 135°, respectively. We can see that when the incident polarization states correspond to the points a, b, c, and d on the conventional PS shown in Figure 3a, respectively, the polarization state of the VBs generated on the focal plane *z*_1_ map to points a_1_, b_1_, c_1_, d_1_ on HOP sphere, whereas those on z_2_ correspond to points c_1_, d_1_, a_1_, b_1_, as shown in Figure 3b.

## 4. Conclusions

In summary, we proposed an HWP metasurface for generating longitudinally separated dual-channel vectorial structured light fields, which consists of two interleaved sub-metasurfaces. In each sub-metasurface, both the propagation and P-B phases are employed to independently construct helical phase profiles for vortex generation, while the propagation phase is also used to yield a hyperbolic phase profile for beam focusing. Moreover, the opposite radial gradient phases are imparted on the two sub-metasurfaces via the P-B phase, leading to the spin separation of LCP and RCP vortices along the propagation direction. Under elliptically polarized illumination, dual-channel HOP or HyOP beams of different orders can be generated on the two focal planes *z*_1_ and *z*_2_, respectively. By varying the ellipticity of the incident light, the propagation-phase topological charge and the rotation order *m*^A/B^, dual-channel vectorial structured lights with polarization states evolving along an arbitrary pair of antipodal meridians on the HOP or HyOP sphere can be obtained. The theoretical derivations and simulation results are presented and are in good agreement. This work demonstrates a novel approach to achieve longitudinally separated multi-channel wavefront manipulation, which shows great promise for multifunctional integrated optical devices and exhibits significant potential in optical micromanipulation, light-matter interaction, and optical communications.

## Data Availability

The data that support the findings of this study are available from the corresponding author upon reasonable request.
